# Mediating effect of family support on the relationship between acculturation and loneliness among the migrant elderly following children in Jinan, China

**DOI:** 10.3389/fpubh.2022.934237

**Published:** 2022-08-17

**Authors:** Di Zong, Zhongqian Lu, Xinfei Shi, Ying Shan, Shixue Li, Fanlei Kong

**Affiliations:** ^1^Centre for Health Management and Policy Research, School of Public Health, Cheeloo College of Medicine, Shandong University, Jinan, China; ^2^NHC Key Lab of Health Economics and Policy Research (Shandong University), Jinan, China

**Keywords:** loneliness, acculturation, family support, migrant elderly following children, mediating effect

## Abstract

The total number of migrant elderly following children (MEFC) has gradually increased along with population aging and urbanization in recent decades in China. The purpose of this study was to investigate the mediating effect of family support on the relationship between acculturation and loneliness among the MEFC in Jinan, China. A total of 656 MEFC were selected by multistage cluster random sampling. Loneliness was measured using the short-form UCLA Loneliness Scale (ULS-8), while acculturation and family support were assessed using a self-designed questionnaire. Descriptive analysis, univariate analysis, and the structural equation model (SEM) were conducted to illustrate the relationship between the above indicators and loneliness. The average ULS-8 score of the MEFC was 12.82 ± 4.05 in this study. Acculturation of the MEFC exerted a negatively direct effect on loneliness and a positively direct effect on family support simultaneously, while family support exerted a negatively direct effect on loneliness. Family support partially mediated the relationship between acculturation and loneliness [95% CI: −0.079 to 0.013, *p* < 0.001], while the mediating effect of family support accounted for 14.0% of the total effect. The average ULS-8 score of 12.82 ± 4.05 implied a low level of loneliness in the MEFC in Jinan, China. Acculturation was found to be correlated with loneliness, while the mediating role of family support between acculturation and loneliness was established. Policy recommendations were provided to reduce loneliness and improve the acculturation and family support of the MEFC according to the findings above.

## Introduction

As the population has aged and urbanization has increased in recent decades, the number of migrant elderly has increased in China (1). According to the data of the Seventh National Population Census of China conducted in 2020, there were 375 million migrants, with a 70% increase seen over the past decade. Among them, 124 million were inter-provincial and 251 million were intra-provincial (2). Meanwhile, the proportion of the elderly among the migrant population in China continually increased from 4.9% in 2000 to 5.3% in 2015 (3). Among the significant number of the migrant elderly in China, those who migrated from their original residence following their children to another city in China in order to take care of the younger generation or to receive healthcare services were defined as the migrant elderly following children (MEFC) (4). The MEFC left their hometowns where they had lived for a long time and migrated to unfamiliar cities. The new living environment of the inflow cities may cause various problems for the MEFC, such as lifestyle changes, shrinkage of social networks, and unfamiliarity with local languages. These problems could also lead to physical discomfort, less social participation, lower acculturation, loneliness, and other mental health problems. The MEFC have thus become a vulnerable group in China's fast economic and social development and deserve more focus and research (5).

Peplau and Perlman suggest the following definition for loneliness: “loneliness is a painful feeling, usually a feeling that one's social needs are not met by the quantity or quality of one's social relationships, especially the quality (6, 7).” According to previous research, the level of loneliness increased with age among the elderly, especially those over 70 years old (8, 9). Tao's research revealed that the MEFC are more likely to feel loneliness in a new environment, which further affects their interpersonal relationships (10). Chinese immigrants in Canada were found to be lonely in Tam and Neysmith's study because their dependent children were unable to provide them with enough emotional support (11). In addition, a study on immigrants to Canada found that second-generation immigrants were lonelier than native-born Canadians (12).

Redfield defined the concept of acculturation as follows: “Acculturation comprehends those phenomena which result when groups of individuals having different cultures come into continuous first-hand contact, with subsequent changes in the original cultural patterns of either or both groups” (13). Studies have shown that the MEFC would face acculturation problems, including language, diet, customs, values, religious beliefs, etc. (14), while low levels of acculturation could further lead to high levels of loneliness (15, 16). Research among elderly immigrants in New Zealand reported that the challenges of loneliness for older immigrants stemmed from problems with culture, language, etc. (17). One study among migrant children in China found that migrant children with low levels of acculturation and social acceptance might experience greater stress and loneliness (18). Although previous studies have explored the relationship between acculturation and loneliness among elderly immigrants and migrant children, there is no research that has examined this association among the MEFC.

The loneliness experienced by the elderly in China is mainly affected by family support from spouses or children, etc. (19). A review by Bai described how harmonious family relationships could enable the MEFC to obtain reliable emotional support and avoid loneliness (20). A further study found that the disabled elderly with high levels of family support had lower levels of loneliness in Tangshan, China (21). It was also found that good family support could lead to better care and could reduce the loneliness of the elderly in Shanghai, China (22). A survey of elderly people in rural Anhui Province, China found that there was a high degree of loneliness, and social and family support played an important role in the development process of loneliness (23). Family support was also confirmed to play an important role in alleviating loneliness among older adults in a study of elderly Malaysians (24). High levels of family support were observed to be capable of preventing the loneliness of patients with cancer in Turkey (25). Although there have been studies on the relationship between family support and loneliness, there are relatively few studies on the MEFC.

Concerning the relationship between acculturation and family support, the existing research has generally found that family support increased with acculturation. Jewell's study revealed that higher acculturation led to higher family support among Mexican American women (26). One study of Chinese American older adults showed that good family support and cohesion could promote their acculturation (27). In addition, previous studies revealed that higher acculturation of children would lead to lower family support of their parents (28). Perez-Brena's research found that it might be easier for young immigrants to integrate into the local area than their parents and they accordingly had a higher degree of acculturation which would lead to differences in cultural values between young immigrants and their parents resulting in lower family support for the migrant elderly (29). Relatively few studies have investigated the association between acculturation and family support and we are not aware of any studies that have examined this relationship among the MEFC.

No research could be found to clarify the relationship between acculturation, family support, and loneliness simultaneously, while more attention has been paid to the relationship between acculturation, family support, and mental health (30). In a study of high school students who migrated to Norway, the cultural impact on mental health was found to be mediated by family support (31). In addition, a study of Chinese American older adults found that acculturation could affect the mental health of older immigrants through family support (32).

In summary, some studies have explored the relationship between acculturation and loneliness, family support and loneliness, and acculturation and family support, yet no research has ever clarified the association between acculturation, family support, and loneliness simultaneously, nor mentioned the MEFC as the research subject. This study aimed to clarify the relationship between acculturation and loneliness, and further verify whether there was a mediating effect exerted by family support on this relationship among the MEFC in Jinan, China.

## Methods

### Study location and its population conditions

Shandong Province is located in the east of China and Jinan is its capital city. Its GDP in 2020 was about 1.01 trillion Yuan (about US $158 billion), an increase of 4.9% over the previous year. As of 1 July 2020, Jinan has jurisdiction over 10 districts, two counties, 132 streets, and 29 towns. By the end of 2020, Jinan had a permanent population of 9.2 million, with an average annual growth rate of 1.27%. Among the permanent residents, the population living in towns is 6.76 million, accounting for 73.46%, and the population living in the countryside is 2.44 million, accounting for 26.54% (33). The seventh census showed that Jinan had a migrant population of 1.83 million (2).

### Data collection and research participants

The data were collected in Jinan, Shandong Province, China in August 2020. The subjects of the study were the elderly over 60 years old who followed their children to Jinan, China. This study adopted the method of multistage cluster sampling to select subjects. In the first stage of data collection, three regions were selected from a total of 10 regions as primary sampling units (PSU), considering the economic development and geographical location of Jinan, China. In the second stage, three zones were selected from each primary sampling unit (PSU) as secondary sampling units (SSU). In the third stage, three communities were selected from each SSU. Then, in these three communities, all elderly people over 60 who migrated with their children were taken as the total sample of this study.

In this survey, a total of 32 college students acted as investigators and received training on research background information, questionnaire content, and social survey skills. The investigators collected data in the form of face-to-face interviews with the subjects for about 20 min each. A total of 670 MEFC were selected and interviewed at first. However, 14 of them were excluded because there were obvious logical errors in their answers or their questionnaires were not completed. A total of 656 MEFC in Jinan, China were ultimately included in the study.

### Culture shock theory

The theoretical basis is based on anthropologist Oberg's Culture Shock Theory. It refers to the sense of loss and accompanying anxiety in the process of cultural transformation. Oberg believes that culture shock is the result of an individual's contact with a different culture, such as tension and anxiety, and it is also the individual's sense of loss, confusion, and incompetence due to separation from the familiar culture and social symbols of the original culture. Studies have shown that culture shock generally goes through four stages, honeymoon stage, depression stage, adjustment stage, and adaptation stage. The change process of these four stages generally takes the form of a “U”-shaped curve. In the third stage, when immigrants gradually understand the difference between the original culture and the foreign culture, and gradually understand the local customs, they begin to adjust their emotions, gradually get out of the painful emotions, and gradually have their own social contact, with certain social support, which will lead to the gradual reduction of psychological loss and loneliness (34). Based on the culture shock theory, a structural equation model between the acculturation, family support, and loneliness of the MEFC was constructed in this study. When the MEFC gradually adapts to the local culture and customs, it will lead to lower loneliness. Meanwhile, acculturation will also result in a good family atmosphere and better family support, and finally, indirectly reduce the loneliness of MEFC.

### Measurements

#### Loneliness

This study used the short-form UCLA Loneliness Scale (ULS-8) to assess the loneliness of the MEFC in Jinan, China. There are a total of eight items in the ULS-8, each item can score 1–4 points (never, rarely, sometimes, always), and the total score is between 8 and 32 points. The eight items were the following: “(1) I lack companionship; (2) There is no one I can turn to; (3) I feel left out; (4) I feel isolated from others; (5) I am unhappy being so withdrawn; (6) People are around me but not with me; (7) I am an outgoing people; (8) I can find companionship when I want it.” The higher the score, the higher the degree of loneliness (35).

#### Acculturation

Four questions were used as the indicators of acculturation: “(1) understanding of local wedding customs; (2) understanding of local funeral customs; (3) understanding of local diet customs; and (4) understanding of local special food snacks.” Two levels of answers were set for each question in the current research: “1. don't understand; 2. understand.”

#### Family support

Family support in this study was measured by four aspects: “(1) children's support, (2) couple's support, (3) siblings' support, and (4) other family members' support.” Two levels of answers were set for each question: “1. no support or low support; 2. high support.”

### Data analysis

A descriptive statistics, *t*-test, and analysis of variance (ANOVA) were employed to describe and determine the statistically significant differences between demographic characteristics, acculturation, and family support by adopting the SPSS 26.0. A *p-*value of < 0.05 was considered to be statistically significant. The structural equation model (SEM) was used to explore the relationship between acculturation, family support, and loneliness among the MEFC in Jinan, China. In this study, maximum likelihood estimation was used to estimate the best fitting model. The SEM consisted of endogenous and exogenous variables, the former being loneliness and the latter being acculturation and family support. The AMOS (IBM, Armonk, New York, USA) statistical software package for Windows was conducted to run the SEM in order to obtain the maximum likelihood estimation of model parameters and to calculate the model fitness index. Finally, we performed bootstrap tests (the sampling process was repeated 1,000 times) to examine the total, indirect, and direct effects of the model. The indirect effect was regarded as statistically significant if the 95% confidence interval (CI) excluded zero (36).

## Results

### General characteristics of the subjects

Table 1 shows the demographic characteristics of the MEFC in this study. A total of 656 MEFC were included in the data analysis, with a mean age of 66.19 ± 4.53 years old. Among them, 30.1% were 63–65 years old. The average score of ULS-6 among the MEFC was 12.82 ± 4.05 in this study. For the sociodemographic information, most of the MEFC were female (63.7%), had a rural Hukou (87.5%), were currently married (88.9%), were unemployed (74.4%), and had a monthly income of 0–1,000 (64.3%). Concerning education, 18.9% of the MEFC had received a high school education or above. Statistically significant differences between education level and loneliness were found among the MEFC in this study.

**Table 1:** Demographic characteristics of the MEFC.

**Variables**	***N*** **(%)**	**Mean score of**	***t*** **/*F***	***p***
		**ULS-8 (SD)**	**value**	
Total	656 (100)	12.82 (4.05)		
Sex			0.608^a^	0.436
Male	238 (36.3)	12.66 (4.14)		
Female	418 (63.7)	12.92 (4.00)		
Age (years)			1.160^b^	0.296
60–62	126 (19.2)	17.01 (2.63)		
63–65	197 (30.1)	17.05 (2.98)		
66–68	183 (27.9)	16.73 (3.08)		
69–	150 (22.9)	17.17 (3.07)		
Hukou			0.364^a^	0.716
Rural	574 (87.5)	12.84 (4.10)		
City	82 (12.5)	12.67 (3.74)		
Marital status			1.592^a^	0.112
Currently married	583 (88.9)	12.73 (4.03)		
Single	73 (11.2)	13.53 (4.16)		
Employment			2.416^b^	0.090
Employed	37 (5.6)	12.62 (4.29)		
Retired	131 (20.0)	12.15 (3.88)		
Unemployed	488 (74.4)	13.02 (4.07)		
Education level			1.765^a^	0.152
Illiteracy	196 (29.9)	13.36 (4.18)		
Primary school	144 (22.0)	12.75 (3.94)		
Junior high school	192 (29.3)	12.55 (4.04)		
High school and above	124 (18.9)	12.48 (3.97)		
Monthly income			0.849^b^	0.630
0–1,000	422 (64.3)	17.11 (3.06)		
1,001–2,000	78 (11.9)	16.17 (2.54)		
2,000 and above	156 (23.8)	16.72 (2.78)		

*MEFC, migrant elderly following children*.

a *t-test*.

b *F-test*.

Table 2 shows the acculturation, family support, and loneliness of the MEFC. In terms of acculturation, the percentage of the MEFC who knew about the wedding customs, funeral customs, diet customs, and special food snacks of the inflow city was 42.4, 40.1, 51.1, and 46.5%, respectively. Concerning family support, most of the MEFC were able to obtain high support from their children (89.5%) and spouse (83.5%), while the percentage of support from siblings and other family members was 53.8 and 36.4%, respectively. Statistically significant differences were observed between loneliness and the four variables of assessing acculturation (wedding customs, funeral customs, diet customs, and special food snacks) separately in this study. Meanwhile, statistically significant differences between loneliness and the three variables of measuring family support (children support, sibling support, and other family support) were also found among the MEFC in the current study.

**Table 2:** Acculturation and Family support of the MEFC.

**Variables**	***N*** **(%)**	**Mean score of**	***t***	***p***
		**ULS-8 (SD)**	**value**	
Wedding customs		12.82 (4.05)	3.749	<0.01
Don't understand	378 (57.6)	17.34 (3.08)		
Understand	278 (42.4)	16.47 (2.72)		
Funeral customs			3.791	<0.01
Don't understand	393 (59.9)	17.33 (3.06)		
Understand	263 (40.1)	16.44 (2.74)		
Diet custom			4.737	<0.01
Don't understand	321 (48.9)	17.53 (3.11)		
Understand	335 (51.1)	16.45 (2.72)		
Special food snacks			4.936	<0.01
Don't understand	351 (53.5)	17.50 (3.05)		
Understand	305 (46.5)	16.37 (2.74)		
Children support			2.270	<0.05
None or low support	69 (10.5)	17.74 (3.09)		
High support	587 (89.5)	16.89 (2.94)		
Spouse support			0.762	0.121
None or low support	108 (16.5)	17.38 (3.14)		
High support	548 (83.5)	16.90 (2.92)		
Sibling support			3.830	<0.01
None or low support	303 (46.2)	17.45 (2.85)		
High support	353 (53.8)	16.57 (3.00)		
Other family support			5.140	<0.01
None or low support	417 (63.6)	17.42 (2.86)		
High support	239 (36.4)	16.21 (2.99)		

*MEFC, migrant elderly following children*.

### Structural equation modeling analysis

#### Model fitness indices

Table 3 displays the value of the model fit indicators of this study. As shown, the information of the model fitness indices was: CFI = 0.946 > 0.90; GFI = 0.938 > 0.90; AGFI = 0.915 > 0.90; RMSEA = 0.061 < 0.080. The chi-square value of the overall model was *p* < 0.001. All of the model fitness indices values indicated that the hypotheses model fits the empirical data very well in this study.

**Table 3:** The model fit index.

**Index**	***p***	**CFI**	**GFI**	**AGFI**	**RMSEA**
Change range	–	0–1	0–1	–	–
Reference standard	*p* < 0.05	>0.90	>0.90	>0.90	<0.080
Actual value	*p* < 0.001	0.946	0.938	0.915	0.061

*CFI, Comparative Fitness Index; GFI, Goodness of Fit Index; AGFI, Adjusted Goodness of Fit Index; RMSEA, Root-mean Square Error of Approximation*.

#### Relationship between acculturation, family support, and loneliness assessed by the SEM

**Figure 1:**
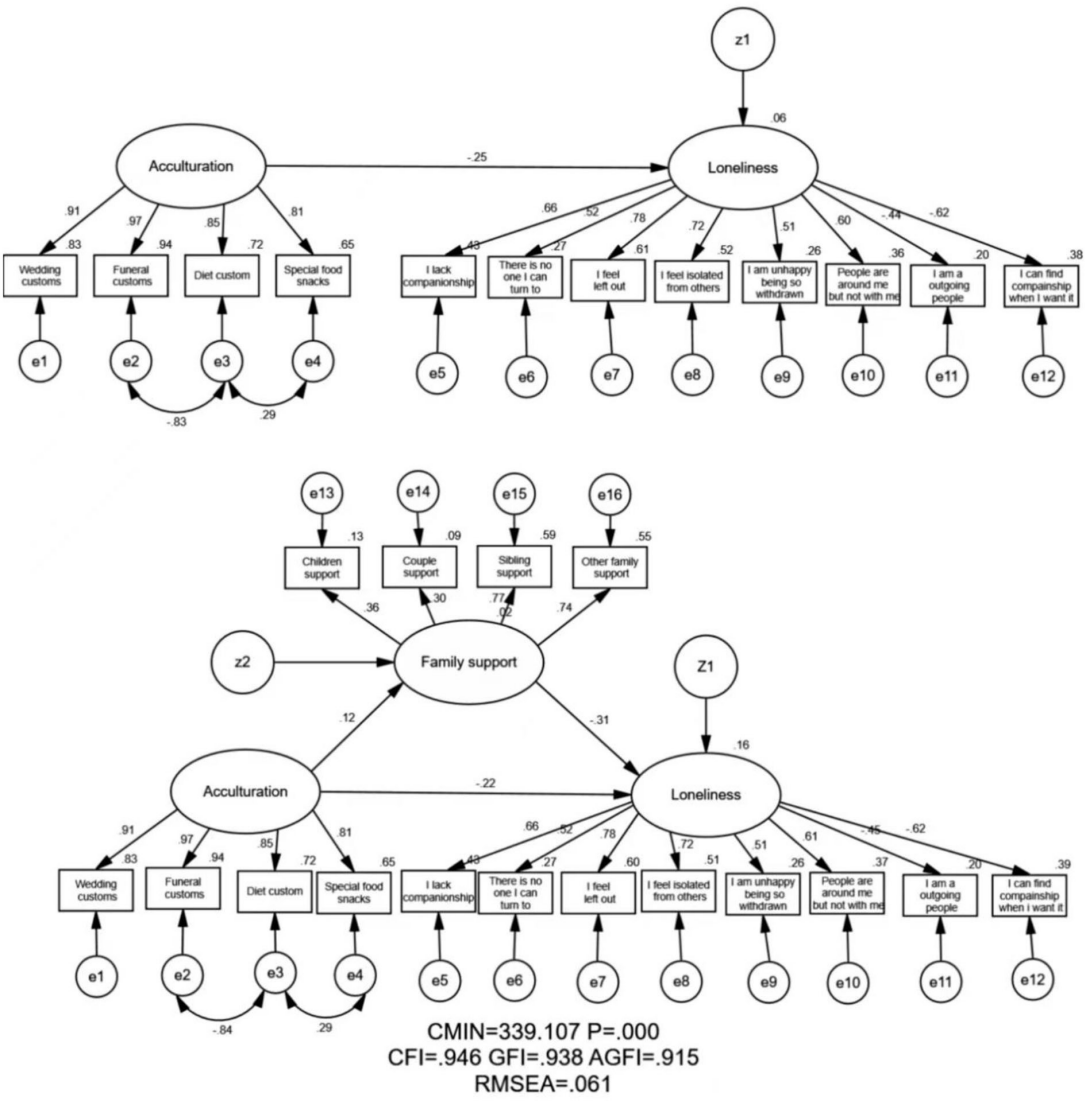
Path diagram of the association between acculturation and loneliness with family support as a mediator (*n* = 656). Employing the cross-sectional data, the relationship between acculturation and family support, and loneliness was analyzed. Arrows indicate the associations and directions between variables, and double curved arrows indicate the correlation between each factor. All parameter estimates were statistically significant (*p* < 0.001); χ^2^ = Chi-square; GFI, Goodness of Fit Index; AGFI, Adjusted Goodness of Fit Index; CFI, Comparative Fitness Index; RMSEA, Root-mean Square Error of Approximation; MEFC, migrant elderly following children.

The relationship between acculturation, family support, and loneliness of the MEFC is shown in Figure 1. In detail, there are two figures in Figure 1: the upper figure shows the relationship between acculturation and loneliness, while the lower figure illustrates the mediating effect of family support on the association between acculturation and loneliness. In addition, the SEM could be used not only to analyze empirical relationships between different variables in the model but also to analyze statistical associations between observed and unobserved variables simultaneously. In this study, there were three unobserved variables: acculturation, family support, and loneliness. Among them, acculturation and family support were both represented by four observation variables, and loneliness was represented by eight observation variables.

As illustrated in Figure 1 (upper figure), acculturation exerted a negative effect on loneliness (the standardized effect = −0.25, *p* < 0.01). Moreover, as shown in Figure 1 (lower figure), acculturation exerted a negative effect on loneliness (the standardized effect = −0.22, *p* < 0.01), family support also exerted a negative effect on loneliness (the standardized effect = −0.31, *p* < 0.01), and acculturation exerted a positive effect on family support (the standardized effect = 0.12, *p* < 0.05).

#### The mediating effect of family support on the association between acculturation and loneliness

Figure 1 also illustrated the mediation pathway model. The path coefficients showed that the total effect of acculturation on loneliness was −0.25 (upper figure in Figure 1). After adding family support (lower figure in Figure 1), the direct effect of acculturation on loneliness was −0.22.

Table 4 shows the standardized total effect, direct effect, indirect effects, as well as the results of the mediating effect. Specifically, the standardized indirect effect coefficient of acculturation on loneliness through family support was −0.037, with a mediating effect of 14% [14% = 0.12 × (−0.31)/0.12 × (−0.31) +(−0.22)]. The bootstrap test suggested that the direct mediating effect *via* family support was −0.037 (95%CI: −0.079 to −0.013, *p* < 0.001). These effects were significant since the 95%CI excluded zero. Therefore, the association between acculturation and loneliness was achieved partly through family support.

**Table 4:** The standardized total, direct, and indirect effects of acculturation on loneliness with family support as mediators (*N* = 656).

**Path**	**Standardized effect value**	**SE**	***p***	**Percent (%)**	**95%CI**	
					**LLCL**	**ULCL**
Direct effect	−0.22	0.041	<0.01	86	−0.298	−0.138
Acculturation→ Loneliness						
Indirect effect	−0.037	0.016	<0.01	14	−0.079	−0.013
Acculturation→ Family support						
Family support→ Loneliness						
Total effect	−0.25	0.041	<0.01	100	−0.341	−0.170

*SE, standard errors; CI, confidence interval; LLCL, lower limits confidence interval; ULCL, upper limits confidence interval*.

## Discussion

### Principal findings and comparison with other studies

Employing the SEM, this study investigated the relationship between loneliness, acculturation, and family support among the MEFC in Jinan, China. The results revealed that the loneliness of the MEFC was associated with their acculturation, while family support mediated the relationship between acculturation and loneliness.

#### Loneliness of the MEFC

The mean score of loneliness among the MEFC in this study was 12.82 ± 4.05, indicating that they had a low level of loneliness. This result is lower than a study of Italian elderly people (13.1 ± 6.9) (37), which might be because most of the MEFC come to the inflow city to take care of their children, so their bodies are relatively healthy, while physical frailty and disability are an important cause of loneliness (38). In addition, the loneliness level of this study was also lower compared to a Nigerian study of retired elderly people (20.31 ± 3.59) (39). It might be because most Nigerian elderly people are not accompanied by their children after retirement (40), and although most of the MEFC did not have a job, they were accompanied by their family members, so they had a lower level of loneliness. However, in a study on the elderly in rural China (11.09 ± 4.59) (41), it was found to have a lower level of loneliness than this study. The possible explanation for this difference was the MEFC were not used to the inflow cities and felt unfamiliar or the differences in socioeconomic status among sample cities.

#### Acculturation and loneliness

A negative relationship between acculturation and loneliness was found among the MEFC in this study, that is, the better the acculturation, the lower the loneliness. The negative effect of acculturation on loneliness in our study was similar to that in the study conducted among older immigrants in Sweden, where it was also found that the migrant elderly were not able to adapt to the culture which could lead to feelings of alienation and non-belonging that triggered experiences of loneliness (42). In previous studies, the immigrants who were able to share a similar language and culture had lower levels of loneliness, while those who did not had higher levels of loneliness (43). The relationship between acculturation and loneliness could also be partially explained by the concept of cultural belonging (44). Although it might be a resource for migrants experiencing two different cultures during their migration, there are also inner conflicts in migrants' sense of belonging. Therefore, in terms of cultural belonging, if cultural attachment and inclusion between two cultures could prevent loneliness, then acculturation stress and cultural conflict could also increase the loneliness of migrants (45). Specifically, for the MEFC in this study, most of them came from rural areas and migrated to cities following their children. The big differences between their hometown and inflow city might cause cultural conflicts for them, and further result in increased loneliness.

#### Family support and loneliness

The relationship between loneliness and family support in the MEFC was negative in this study, which was consistent with previous research that found that the level of family function and social support were important factors affecting the loneliness of the elderly (46, 47). Family relationship was an important part of the social network, and children were important sources of companionship, intimacy, and sharing (48). Spouse's company was also an important factor in reducing loneliness among older adults (49). In addition, sibling support could ease the loneliness of older adults (50). It is well-known that Asian populations are characterized by collectivism compared to the individualism of the West (51). Studies have shown that the loneliness of collectivist societies is higher than that of individual socialist societies (52) because the core of collectivism is interdependence which emphasizes the relationship between individuals (53). Srinivasan's research found that strong relationships were the norm in rural areas in India, while a lack of such social connections was akin to a departure from cultural norms and could be seen as painful which finally led to an increase in loneliness (54). In a study on Chinese American older adults, a supportive network was observed to reduce the effects of adverse stressors and to improve mental health outcomes (32). Regarding the MEFC in the current study, their support from family members might help them to face unfamiliar environments, integrate into the inflow city, and reduce their loneliness.

#### Acculturation and family support

A positive relationship between acculturation and family support was observed in this study, implying that the improvement of acculturation would lead to higher levels of family support. This result was consistent with a study on Mexican Americans which also found that those who approved more of Mexican culture and customs would enjoy a higher level of family support (55). Moreover, it was found that second-generation Mexican Americans were more likely to have large family networks and receive family support than first-generation Mexican American women (56). However, a study on Latino-American adolescent boys showed that higher acculturation could lead to family conflicts. This might be due to the fact that the teenagers could more easily and actively integrate into the local culture and environment, while their parents would generally follow the original cultural values and customs. This gap in the acculturation of children and parents might lead to their differences in cultural values, and further cause conflicts among family members (57). For the MEFC, when they have better acculturation, they might become more cheerful, be more willing to communicate with their family members, and be more likely to have a higher level of family support.

#### The mediating effect of family support

This study found that the family support of the MEFC played a mediating role between acculturation and loneliness, and the mediating effect accounted for 14% of the total effect. This was similar to a previous study among Latino adults, which also found that family support had a mediating effect between acculturation and mental health (58). The reason may come from the fact that social support could reduce the stress caused by unfamiliar cultural environments during the intercultural transition of migrants and could further promote their physical and mental health (59). In detail, as an important resource for immigrants (60), social support has been shown to be associated with acculturation (61) and is helpful for the maintenance of the mental health of immigrants (62). Therefore, family support became a mediator between acculturation and loneliness. Compared with the young people who need to develop their careers in the inflow city, the MEFC might not have an urgent need to expand their network and comprehensively adapt to the local culture, since their main job is to take good care of their grandchildren. Moreover, taking care of their grandchildren consumed most of their time and energy, which also caused them to have limited time and energy for purposely enlarging their network and caring for their own health conditions. All of these factors made the MEFC more reliant on family support for adapting to life in the inflow city. In this sense, as a mediating variable, family support played an important role between acculturation and loneliness for the MEFC.

### Implications

The following policy recommendations are provided based on the results of this study: First, the community could arrange certain programs and activities to help the MEFC to learn the culture of the inflow city, to better adapt to local life, and to enhance their sense of belonging. Second, the family members of the MEFC should pay attention to the mental health of the MEFC, provide them with a good living environment, and create a good family atmosphere. Third, community health centers are encouraged to pay attention to the mental health of the MEFC and conduct psychological counseling and health education.

### Limitations

This study has the following limitations: First, cross-sectional data was used and so causality cannot be predicted (such as the association between acculturation and family support). Second, due to the lack of systematic scales of family support and cultural adaptation in the questionnaire, we only selected certain relevant indicators for evaluation, which are expected to be improved in follow-up research. Third, multi-stage cluster sampling was employed to select the participants, yet the survey weights were not applied and calculated. Fourth, more confounding variable effects (such as duration of migration) on the loneliness of MEFC are needed to be examined in future studies. Finally, due to the COVID-19 pandemic, this study only selected the MEFC in Jinan as the research subjects as more surveys in other cities in China could not be conducted. This means that the results cannot represent more MEFC populations in China.

## Conclusion

The average ULS-8 score of 12.82±4.05 indicated a low level of loneliness among the MEFC in Jinan, China. Acculturation was found to be associated with loneliness, while family support was observed to play a partial mediating role between acculturation and family support among the MEFC in Jinan. Policy implications were provided to decrease loneliness and improve the mental health status of the MEFC based on the findings of this study.

## Data availability statement

The original contributions presented in the study are included in the article/supplementary material, further inquiries can be directed to the corresponding author/s.

## Ethics statement

Ethical review and approval was not required for the study on human participants in accordance with the local legislation and institutional requirements. The patients/participants provided their written informed consent to participate in this study.

## Author contributions

DZ analyzed the data and drafted the manuscript. ZL and XS participated in the questionnaire survey and data processing. YS gave many valuable suggestions in response to the reviewer's comments. FK applied for funds to support this study, designed the study, completed the questionnaire design, supervised and participated in the data collection, instructed the writing, statistical analysis, data processing, and provided comments on the modification of the manuscript. SL gave many valuable comments on the draft and also polished it. All authors read and agreed to the published version of the final manuscript.

## Funding

This study was supported and funded by the National Natural Science Foundation of China (No. 71804094), the China Postdoctoral Science Foundation (No. 2016M592161), the Natural Science Foundation of Shandong Province (No. ZR2016GB02), the Postdoctoral Science Foundation of Shandong Province (No. 201603021), and the Fundamental Research Funds of Shandong University (Nos. 2015HW002 and 2018JC055).

## Conflict of interest

The authors declare that the research was conducted in the absence of any commercial or financial relationships that could be construed as a potential conflict of interest.

## Publisher's note

All claims expressed in this article are solely those of the authors and do not necessarily represent those of their affiliated organizations, or those of the publisher, the editors and the reviewers. Any product that may be evaluated in this article, or claim that may be made by its manufacturer, is not guaranteed or endorsed by the publisher.

## Abbreviations

MEFC: Migrant elderly following children
ULS-8: the short-form UCLA Loneliness Scale (ULS-8) the short-form UCLA Loneliness Scale
SEM: structural equation model
χ^2^: Chi-square
GFI: Goodness of Fit Index
AGFI: Adjusted Goodness of Fit Index
CFI: Comparative Fitness Index
RMSEA: Root-mean Square Error of Approximation
SE: standard errors
CI: confidence interval
LLCL: lower limits confidence interval
ULCL: upper limits confidence interval.
